# The complete plastome of tropical fruit *Garcinia mangostana* (Clusiaceae)

**DOI:** 10.1080/23802359.2017.1390406

**Published:** 2017-10-18

**Authors:** Sangjin Jo, Hoe-Won Kim, Young-Kee Kim, Jung-Yeon Sohn, Se-Hwan Cheon, Ki-Joong Kim

**Affiliations:** Division of Life Sciences, Korea University, Seoul, Korea

**Keywords:** Plastome, gene and intron losses, small inversition, *Garcinia mangostana*, Clusiaceae

## Abstract

The complete plastome sequence of *Garcinia mangostana* L. (Clusiaceae) is completed in this study (NCBI acc. no. KX822787). This is a first complete plastome sequence from the Clusiaceae. The complete plastome size is 158,179 bp in length and consists of a large single copy of 86,458 bp and a small single copy of 17,703 bp, separated by two inverted repeats of 27,009 bp. The *G. mangostana* plastome shows four minor structural modifications including *inf*A gene loss, *rpl*32 gene loss, *ycf*3 gene intron loss and a 363 bp inversion between *trn*V-UAC and *atp*E gene. The plastome contains 111 genes, of which 77 are protein-coding genes, 30 are tRNA genes and four are rRNA genes. The average A-T content of the plastome is 63.9%. A total of 110 simple sequence loci are identified from the genome. Phylogenetic analysis reveals that *G. mangostana* is a sister group of *Erythroxylum novogranatense* (Erythroxylaceae) with 78% bootstrap support.

*Garcinia mangostana* L. is commonly known as ‘the queen of fruits’ or mangosteen. It is one of the most delicious tropical fruits originated in Malaysia and Indonesia (Kim 2011). It belongs to the family Clusiaceae of Malpighiales (APG IV 2016). The family Clusiaceae consists of 13 genera and approximately 750 species including many fruit crops (Christenhusz and Byng 2016). But, the complete plastome sequence is not known from the family. Therefore, we first report the complete plastome of *G. mangostana* (Clusiaceae) in this study. It will be a useful reference for the phylogenetic and evolutionary studies of Malphigiales.

The leaves of *G. mangostana* used in this study were collected from the Korea University greenhouse, where we grew the plants from seeds originally collected from Thailand. A voucher specimen was deposited in the Korea University Herbarium (KUS acc. no. 2014-0243). Fresh leaves were ground into powder in liquid nitrogen and total DNAs were extracted using the cetyl trimethyl ammonium bromide (CTAB) method (Doyle and Doyle 1987). The DNAs were further purified by the ultracentrifugation and dialysis (Palmer 1986). The genomic DNAs are deposited in the Plant DNA Bank in Korea (PDBK acc. no. 2014-0243). The complete plastome sequence was generated using an Illumina HiSeq 2000 system (Illumina Inc., San Diego, CA). An average coverage of sequences was 346 times of its annotated plastome size. Annotations were performed using the National Center for Biotechnology Information (NCBI) BLAST and tRNAscan-SE programmes (Lowe and Eddy 1997).

The gene order and structure of the *G. mangostana* plastome are similar to those of a typical angiosperm (Shinozaki et al. 1986; Kim and Lee 2004; Yi and Kim 2012) except four minor modifications. The *inf*A and *rpl*32 genes were lost in the *G. mangostana* plastome. The two gene losses were also reported from other Malpighialian families (Tangphatsornruang et al. 2011; Huang et al. 2014; Bardon et al. 2016; Cheon et al. 2017). The *ycf*3 gene usually consists of three exons and two introns in angiosperm. But, the second intron was lost in *G. mangostana* plastome. The *G. mangostana* plastome had a small 363 bp inversion between *trn*V-UAC and *atp*E region. The palindromic repeats of 15 bp (ACATCCTATTTCTTT/AAAGAAATAGGATGT) were located on the two break points of inversion. This is a new inversion report, but there are several reports of other small inversions due to palindromic repeats (Kim and Lee 2004; Catalano et al. 2009).

The complete plastome is 158,179 bp in length and consists of a large single copy (LSC) region of 86,458 bp and a small single copy (SSC) region of 17,703 bp separated by two inverted repeats (IR) of 27,009 bp. The plastome comprises 111 unique genes (77 protein-coding genes, 30 tRNA genes and four rRNA genes). Seven protein-coding, seven tRNA and four rRNA genes are duplicated in the IR regions. The average A-T content of the plastome is 63.9%, whereas that in the LSC, SSC and IR regions is 66.5%, 69.8% and 57.8%, respectively. Seventeen genes have one intron and one gene (*clp*P) has two introns. A total of 110 simple sequence repeat (SSR) loci are scattered among the plastome. Among these, 88, 15 and 7 are mono-SSR, di-SSR and tri-SSR loci, respectively.

To validate the phylogenetic relationships of *G. mangostana* in rosids, we constructed a maximum likelihood (ML) tree by using 40 super-rosids taxa. Phylogenetic analysis was performed on a data set that included 78 protein-coding genes and four rRNA genes using RAxML version 7.7.1 (Stamatakis et al. 2008). The 82 gene sequences (77,445 bp in length) were aligned with the MUSCLE programme using Geneious version 6.1.8 (Biomatters Ltd.; Kearse et al. 2012). As a result, *G. mangostana* forms a clade with *Erythroxylum novogranatense* (Erythroxylaceae) with a 78% bootstrap value (Figure 1). In previous phylogenetic studies, Clusiaceae belongs to the clusioids group and has been known to be the closest relationship to Bonnetiaceae (Wurdack and Davis 2009; Ruhfel et al. 2011; Xi et al. 2012). There are 36 families in Malphigiales. But, the complete plastomes were reported from only six families so far. In order to solidify the interfamilial relationships in Malphigiales, we need a complete plastome sequences from other families.

**Figure 1.**
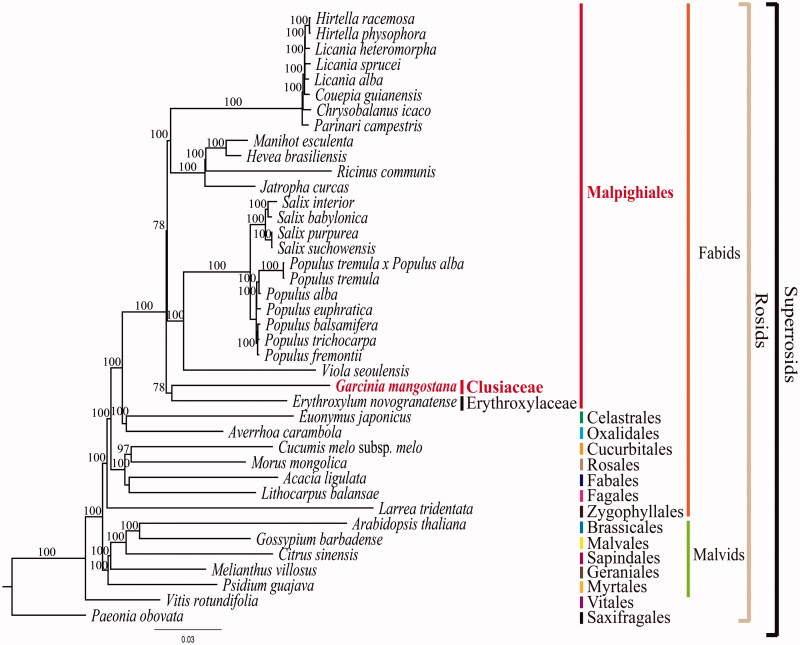
Maximum Likelihood (ML) tree based on 78 protein-coding and four rRNA genes from 40 plastomes as determined by RAxML(−ln *L =* −466117.224833). The numbers at each node indicate the ML bootstrap values. GenBank accession numbers of taxa are shown below, *Acacia ligulata* (NC_026134), *Arabidopsis thaliana* (NC_000932), *Averrhoa carambola* (NC_033350), *Chrysobalanus icaco* (NC_024061), *Citrus sinensis* (NC_008334), *Couepia guianensis* (NC_024063), *Cucumis melo* subsp. *melo* (NC_015983), *E. novogranatense* (NC_030601), *Euonymus japonicus* (NC_028067), *G. mangostana* (KX822787), *Gossypium barbadense* (NC_008641), *Hevea brasiliensis* (NC_015308), *Hirtella physophora* (NC_024066), *H. racemose* (NC_024060), *Jatropha curcas* (NC_012224), *Larrea tridentata* (NC_028023), *Licania alba* (NC_024064), *L. heteromorpha* (NC_024062), *L. sprucei* (NC_024065), *Lithocarpus balansae* (NC_026577), *Manihot esculenta* (NC_010433), *Morus mongolica* (NC_025772), *Melianthus villosus* (NC_023256), *Paeonia obovata* (NC_026076), *Parinari compestris* (NC_024067), *Populus alba* (NC_008235), *P. balsmifera* (NC_024735), *P. euphratica* (NC_024747), *P. fremontii* (NC_024734), *P. tremula* (NC_024725), *P. tremula* × *P. alba* (NC_028504), *P. trichocarpa* (NC_009143), *Psidium guajava* (NC_033355), *Ricinus communis* (NC_016736), *Salix babylonica* (NC_028350), *S. interior* (NC_024681), *S. purpurea* (NC_026722), *S. suchowensis* (NC_026462) *Viola seoulensis* (NC_026986) and *Vitis rotundifolia* (NC_023790).
